# Genetic evaluation of reproduction performance of Jersey × Red Sindhi crossbred cows

**DOI:** 10.14202/vetworld.2016.1012-1017

**Published:** 2016-09-26

**Authors:** S. Vinothraj, A. Subramaniyan, R. Venkataramanan, Cecilia Joseph, S. N. Sivaselvam

**Affiliations:** 1 Department of Animal Genetics and Breeding, Madras Veterinary College, Vepery, Chennai, Tamil Nadu, India; 2 Department of Animal Genetics and Breeding, Post-graduate Research Institute in Animal Sciences, Kattupakkam - 603 203, Chennai, Tamil Nadu, India; 3 Department of ARGO, Madras Veterinary College, Vepery, Chennai, Tamil Nadu, India

**Keywords:** genetic parameters, Jersey crossbred, reproductive performance

## Abstract

**Aim:**

The present investigation was undertaken to study the reproduction performance and effect of non-genetic factors on reproduction performance of Jersey crossbred cows.

**Materials and Methods:**

Data on 355 Jersey crossbred cattle maintained at the Post-graduate Research Institute in Animal Sciences, Kattupakkam, Tamil Nadu, distributed over 30 years (1985 to 2014). The effect of various non-genetic factors including the period of birth, season of birth, period of calving, season of calving and parity were analyzed through least-squares analyses using univariate general linear model. The different (co)variance components for calculation of genetic parameters were estimated using restricted maximum likelihood method by fitting an animal model.

**Results:**

The overall least-squares means (±standard error) of age at first service, age at first calving, weight at first calving, service period, calving interval, dry period, and number of services per conception were 848.06±9.72 days, 1204±12.20 days, 289.81±1.71 kg, 210.01±6.41 days, 489.12±6.45 days, 137.96±5.58 days, 2.50±0.07, respectively. Period of calving had either significant (p<0.05) or highly significant (p<0.01) effect on all reproduction traits studied except service period, calving interval, and dry period. Number of services per conception was affected by season of calving. Parity had significant influence (p<0.05) or highly significant (p<0.01) influence on all the traits studied except service period and dry period. Heritability estimates of age at first service, age at first calving, weight at first calving, service period, calving interval, dry period, and number of services per conception were 0.299, 0.220, 0.017, 0.142, 0.222, 0.177, and 0.042, respectively. The estimates of repeatability for service period, calving interval, dry period, and number of services per conception were 0.219, 0.234, 0.420, and 0.001, respectively.

**Conclusions:**

The reproduction performances of Jersey × Red Sindhi crossbreds were lower when compared to the earlier reports on Jersey crossbreds. Heritability and repeatability values were also low to moderate, indicating limited scope for improvement through selection.

## Introduction

India possesses the largest cattle and buffalo population in the world, but average milk production per animal is very low in comparison with advanced countries. There are 190.9 million cattle and 108.7 million buffaloes in India according to 19^th^ All India Livestock Census [1]. India produced 132.4 million metric tons of milk in 2012-13 and ranked first in the world. In Tamil Nadu, there are 111.89 lakh cattle and 20.09 lakh buffaloes [1]. They produce 69.68 lakhs metric tons of milk in a year. The national per captia requirement is 299 g/day as per the Indian Council of Medical Research recommendation. However, the per captia availability of milk is 240 g in India, and it is 237 g in Tamil Nadu. It is, therefore, necessary to increase the milk production in our country so as to meet the demand. A sustainable dairy industry requires the development of high yielding cows and buffaloes not only to satisfy the demand and supply of milk for the populations. Crossbreeding of indigenous cattle with high yielding exotic breeds has emerged as an effective and suitable strategy to increase milk production. Research on crossbreeding all around the world particularly in the tropical countries has shown that performance of the F_1_ animals is the best with respect to various productive as well as reproductive traits. However, assessment of reproductive performance depends on many parameters to assess overall performance of the dairy animal. However, studies on the reproductive performance of Jersey crossbreds are very limited and restricted only to certain northern and western parts of the country. Varaprasad *et al*. [2] had observed the reproductive performance of Jersey × Sahiwal cows in Andhra Pradesh. It is a long-felt need of the breeders to elucidate the reproduction performance of Jersey crossbreds.

This study will be useful to suggest suitable selection programs, and breeding strategies along with management practices for Jersey crossbred cattle under the climatic conditions prevailing in the north-eastern agro-climatic zone of the state.

To evaluate the reproductive performance of Jersey crossbred cattle in North-eastern agro-climatic zone of Tamilnadu and estimate genetic parameters for various reproductive traits.

## Materials and Methods

### Ethical approval

Ethical approval was not necessary for this study as no experimental animals were used.

### Study area

The present study was carried out at the Post-graduate Research Institute in Animal Sciences (PGRIAS), Kattupakkam, Chennai, Tamil Nadu, India. It is located at 12.5°N latitude and 80.07°S longitudes, and this region is generally experiences hot and humid climatic conditions. The farm receives rain under the influence of both southwest and northeast monsoons. The farm gets an average annual rainfall of 1159 mm in about 47-60 rainy days. Relative humidity ranges between 58% and 84%. Relative humidity is maximum in the morning (74%) and minimum in the evening (64%). Higher values of 83-84% are observed between November and January. The minimum and maximum temperatures are 21.5°C and 41°C, respectively.

### Herd structure

In this farm, crossbreeding program was started at PGRIAS, in 1971, using Jersey and Red Sindhi germplasm with an objective of developing Jersey crossbred animals suitable to the agro-climatic conditions prevailing in Tamil Nadu. During the period of study, *inter-se* mating of 50% Jersey × Red Sindhi crossbred cattle is practiced.

### Management

The Jersey × Red Sindhi crossbred cows are maintained under semi-intensive system of management. Napier-bajra hybrid grass (CO_3_) is cultivated on the farm to ensure green fodder supply throughout the year. The green fodder is chaffed and fed at the rate of 15-20 kg per adult animal. The adult animals are also allowed to graze in the field after milking. Concentrate mixture is provided as per the standard requirements at the rate of 1.5 kg for maintenance and additional 500 g for every 1 kg of milk produced and is fed in equally divided quantities before milking. Calves are weaned at birth and pail feeding is practiced. Calves are fed with colostrum for the first 5 days and are pail fed with milk up to 30 days. Then, the quantity of milk is reduced with the gradual inclusion of calf starter in the ration. In addition, nibbling of green legume and tender grass is also encouraged. Artificial insemination is used for breeding the cows. Heifers which attain two-thirds of the adult body weight (200 kg) or 18 months of age are inseminated. Animals are inspected for estrus signs and animals in estrus are inseminated 12 h after the onset of estrus, and insemination is repeated at 24 h after onset of estrus, if necessary. Health cover included vaccination against foot and mouth disease at 6 months of age, and it is repeated at 6 months interval. All the animals are dewormed regularly as per the recommended schedule. Calves are identified by tattooing and adult animals by ear tagging. Cows are hand-milked twice approximately at 12 h interval between milkings. Periodical culling of animals is done, and they are sold by auction. The common reasons for culling are disease, poor production, and reproduction performance.

### Standardization of records

A total of 355 animals and 732 overall lactation records were available. Abnormal lactations due to abortion, stillbirth, premature birth, and mastitis were eliminated from the study. Incomplete records due to disposal or death of animals were excluded. The records of animals with <100 days of lactation length were also discarded.

### Traits considered

The reproductive traits studied were age at first service: Age of the heifer at first service expressed in days; age at first calving: Age of the heifer at first calving expressed in days; weight at first calving: Weight of the heifer at first calving expressed in kg; service period: Period in days between calving and subsequent service resulting in fruitful conception; calving interval: Interval between two successive calvings expressed in days; dry period: The duration in days from the date of drying to the next initiation of lactation; number of services per conception: It is a measure of fertility of a herd, the number of services required to effect pregnancy.

### Statistical analysis

Least-squares method in a general linear model was used to study the effect of various genetic and non-genetic factors on various traits. The factors studied were a period of birth, season of birth, period of calving, season of calving and parity. Covariance components were estimated through restricted maximum likelihood technique using an animal model. The fixed effects that were found to be significant from the least-squares analyses were fitted for each trait. The direct genetic effect was fitted as random effect in addition to the significant fixed effects for analysis of first lactation traits. The permanent environmental effect was fitted as an additional random effect for overall lactation traits. All the analyses were carried out using the WOMBAT software. The genetic parameters calculated from the model were used to estimate breeding values for various traits using best linear unbiased prediction. The least-squares means were obtained using the mixed models given below:

### Age at first service and calving

Y_ijk_=μ+p_i_+s_j_+e_ijk_

Where, Y_ijk_=Age at first service and calving of k^th^ heifer/cow, born/calved in the i^th^ period, and j^th^ season, μ=Overall mean when equal subclass frequencies exist, p_i_=Effect of i^th^ period (i=1-5), s_j_=Effect of j^th^ season (j=1-4), and e_ijk_=Random errors normally and independently distributed with mean zero and variance [NID (0, 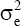)].

### Reproductive traits

Y_ijk_=m+p_i_+s_j_+b(A_ijk_)+e_ijk_

Where, Y_ijk_=First lactation character of the k^th^ cow that calved in the i^th^ period and j^th^ season, μ=Overall mean when equal subclass frequencies exist, p_i_=Effect of i^th^ period (i=1-5), s_j_=Effect of j^th^ season (j=1-4), b=Partial regression of the first lactation trait (Y) on age at first calving (A), A_ijk_=Age at first calving for the corresponding Y_ijk_ observation, and e_ijk_=Random errors NID (0,).

## Results and Discussion

### Effect of non-genetic factors

Period of birth had no significant influence on the age at first calving but had a significant effect on the age at first service in the present study. Similarly, the significant influence of the period of birth on age at first calving and age at first service were reported by Rahumathulla *et al*. [3], Demeke *et al*. [4], and Akhtar *et al*. [5]. Non-significant influence of the period of birth on age at first calving in the present study indicates that the management practices were, by and large, similar during periods indicated in the study. Season of birth did not influence either the age at first calving or age at first service. This is contrary to the findings of earlier studies on Jersey crossbred cows [2,3]. The animals born during autumn were the youngest at first calving. This could be due to better conception rate as a result of the better growth of the calf. Period of calving did not influence (Table-1) any of the reproductive traits studied. These results are agreement with findings of Subramanian and Ulaganathan [6] and Banda [7]. However, this is contrary to the findings of earlier studies on Jersey crossbred cows of Demeke *et al*. [4] and Hadge *et al*. [8]. Season of calving had no significant influence (Table-1) on the reproductive traits except number of services per conception which is comparable with the findings of the earlier reports [6,8].

**Table-1:** Least-squares means (±SE) for service period (days), calving interval (days), dry period (days), and number of services per conception in Jersey×Red Sindhi crossbred cows.

Effect	Service period (days)		Calving interval (days)		Dry period (days)		Number of services per conception	
			
	n	Mean±SE	n	Mean±SE	n	Mean±SE	n	Mean±SE
Overall mean (μ)	502	210.01±6.41	522	489.12±6.45	456	137.96±5.58	729	2.50±0.07
Period of calving	-	NS	-	NS	-	NS	-	NS
P1 (1988-92)	105	199.81±15.66	107	480.49±16.04	86	147.20±15.67	129	1.93±0.12
P2 (1993-97)	127	222.13±12.12	127	507.24±12.74	133	153.24±10.76	212	2.31±0.11
P3 (1998-02)	69	220.43±18.44	81	502.43±17.94	58	123.32±14.52	131	3.17±0.18
P4 (2003-07)	95	226.34±15.73	99	499.09±15.11	83	132.79±12.40	124	2.54±0.14
P5 (2008-14)	106	184.98±11.15	108	457.23±10.66	96	121.84±09.47	133	2.67±0.15
Season of calving	-	NS	-	NS	-	NS	-	**
Winter (Dec-Feb)	146	209.90±12.76	152	492.20±13.10	126	146.54±12.54	209	2.39±0.12^b^
Summer (Mar-May)	115	242.46±13.78	121	515.66±12.94	108	156.76±11.84	164	2.65±0.14^ab^
Rainy (Jun-Aug)	96	204.88±14.04	97	478.22±13.99	86	113.24±09.99	133	2.89±0.16^a^
Autumn (Sep-Nov)	145	188.36±10.75	152	471.86±11.44	136	130.72±09.14	223	2.27±0.11^b^
Parity	-	NS	-	**	-	NS	-	*
First	209	246.45±11.20	224	518.12±10.99^a^	201	160.64±09.80	307	2.20±0.09^b^
Second	132	196.26±11.51	140	481.85±11.75^ab^	120	129.94±09.36	216	2.75±0.13^ab^
Third	87	180.87±11.87	91	462.86±12.64^ab^	78	116.75±11.00	104	2.53±0.17^b^
Fourth	44	169.90±17.29	45	449.26±17.46^ab^	37	118.64±15.99	61	2.77±0.2^ab^
Fifth	21	185.23±25.03	13	476.46±35.50^ab^	12	085.50±19.00	30	2.76±0.33^ab^
Sixth and above	9	110.44±10.51	9	363.88±12.27^b^	8	063.50±03.39	11	3.54±0.62^a^

* p<0.05. Means with at least one common superscript within classes do not differ significantly (p>0.05). n=Number of observations, NS=Not significant, SE=Standard error

### Least-squares means

The mean age at first service observed (848.06±9.72 days) for the present study was lower than the reports of Maurya and Saraswat [9] but higher than Varaprasad *et al* [2]. The higher age at first service is indicative of delay in maturity which might be due to management lapses and environmental conditions. Age at first calving is another important economic character, which influences the productive period and lifetime milk production. The mean age at first calving estimated (Table-2) in the present study was higher (1204.00 days) than the most of the earlier reports by Varaprasad *et al*. [2], Demeke *et al*. [4], Subramanian and Ulaganathan [6], and Maurya and Saraswat [9], and the mean service period is in close agreement with the results of Subramanian and Ulaganathan [6] for Jersey crossbreds reared at the same location. Most of the earlier reports Demeke *et al*. [4] and Hussain *et al*. [10] were revealed lower mean values for service period for pooled lactations than the mean of the present study. The longer service period is again indicative of substandard reproductive performance due to poor adaptation of the herd.

**Table-2:** Least-squares means (±SE) for age at first service (days), age at first calving (days), and weight at first calving (kg) in Jersey×Red Sindhi crossbred cows.

Effect	Age at first service (days)		Age at first calving (days)		Weight at first calving (kg)	
		
	n	Mean±SE	n	Mean±SE	n	Mean±SE
Overall mean (μ)	307	848.06±9.72	313	1204.00±12.20	311	289.81±1.71
Period of birth	-	*	-	NS	-	NS
P1 (1985-89)	90	898.54±16.29^a^	72	1260.47±25.30	91	283.52±3.35
P2 (1990-94)	80	863.52±20.12^ab^	86	1155.24±24.06	95	295.95±2.39
P3 (1995-99)	43	826.18±28.04^bc^	61	1243.86±27.02	65	292.13±2.68
P4 (2000-04)	48	786.45±26.60^c^	51	1152.19±28.05	33	284.54±7.62
P5 (2005-11)	46	807.13±17.80^bc^	43	1211.83±30.58	27	290.25±7.33
Season of birth	NS	NS	-	NS	-	NS
Winter (Dec-Feb)	70	858.10±16.99	67	1239.40±23.22	64	292.73±3.95
Summer (Mar-May)	67	873.65±22.14	73	1235.49±22.44	68	288.91±3.45
Rainy (Jun-Aug)	67	829.53±18.88	69	1178.82±25.60	67	291.38±3.98
Autumn (Sep-Nov)	103	836.64±18.66	104	1175.78±23.96	112	287.75±2.72

* p<0.05, Means with at least one common superscript within classes do not differ significantly (p>0.05). n=Number of observations, NS=Not significant, SE=Standard error

Calving interval is an important trait in lactating animals, which influences the economic productive span of dairy animal. The mean pooled overall calving values calculated (Table-1) are higher than the earlier studies [4,9,10] for calving interval of Jersey crossbred cows. It was lower than the value reported by Mondal *et al*. [11]. The mean calving interval in the present study is much longer than that would be optimal to obtain annual calf crops. Longer calving interval with increasing trend results in poor economic efficiency from animal production.

The dry period is an important resting period for the dairy cow when fresh udder tissue is formed in readiness for the ensuing lactation. The mean value estimated for pooled over lactations dry period in the present study was 137.96 days is almost similar to the report of Haider *et al*. [12] and longer than the earlier report on Jersey crossbred cows [7]. In dairy animal, number of services per conception is the most important factors which directly affect the calving interval. In the present investigation, the mean number of services required per conception was 2.50 in overall lactations. These values are higher than those reported in all earlier studies Mondal *et al*. [11], Akhtar *et al*. [5], and Haque *et al*. [13]. The lowest value of 1.07 was observed, and the highest value of 1.78 was recorded in Jersey × Sahiwal cows [5,12]. The number of services per conception depends on various factors such as quality of semen, state of reproductive system of the female, efficient heat detection, time of insemination, skill of the inseminator, and other management factors. Finally, agro-climatic conditions play a key role in conception.

### Genetic parameters

#### Heritability

The heritability estimate of age at first service was moderate (0.299±0.13) in the present study. Moderate heritability implied that age at first service could be shortened in subsequent generations by selection based on the individual performances. The heritability of age at first calving obtained for Jersey × Red Sindhi was 0.220±0.11, and this was higher than the values reported by Singh *et al*. [14] and Bajetha and Singh [15] but lower than that of Dubey and Singh [16]. Therefore, the heifers delivered the first calf at an earlier age, compared to others could be used for the improvement of the trait. This is important because a shorter age at first calving results in a higher chance for the harvesting of more calves and longer production lives. Service period is a component trait of calving interval, and hence, directly influences the length of the calving interval. The heritability estimate of service period obtained for the herd under study was 0.142±0.09 for pooled over lactations (Table-3). The low heritability value for service period, being a reproductive trait is not unusual. However, higher estimates for service period were reported in an earlier study on Jersey × Sahiwal crossbreds [16].

**Table-3:** Estimates of (co) variance components and genetic parameters for pooled lactation traits in Jersey×Red Sindhi crossbred cows.

Trait	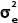	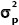	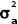	h^2^±SE	r±SE
Reproduction traits					
Age at first service	20589.4	29039.6	8650.1	0.299±0.13	-
Age at first calving	34527.1	44267.3	9740.2	0.220±0.11	-
Weight at first calving	841.57	856.13	14.56	0.017±0.09	-
Service period	14559	18643.3	2648.2	0.142±0.091	0.219±0.06
Calving interval	15552.7	20317.6	4518.43	0.222±0.101	0.234±0.06
Dry period	8098.11	13974.9	2468.39	0.177±0.115	0.420±0.06
Number of services per conception	2.92	3.0533	0.126	0.042±0.003	0.001±0.00

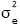
 =Error variance, 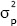 =Phenotypic variance, 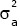 =Additive genetic variance, h^2^=Heritability, r=Repeatability, SE=Standard error

The heritability estimates of 0.222±0.101 for calving interval of Jersey × Red Sindhi herd are lower than the earlier report [1]. Calving interval is influenced more by environmental factors and sufficient improvement in the trait could be brought about by management interventions. The first calving interval was more important as it is available early in life of the animal which could be used for selection. Dry period is also a component trait of the calving interval and is likely to possess little additive genetic variance. As expected the estimated heritability obtained was 0.177±0.115 for pooled over lactations in Jersey × Red Sindhi crossbred cows, and the values are much lower than the estimates obtained in earlier studies [1,8]. The low heritability estimate for dry period indicates that dry period is probably not under the influence of additive gene action and is an environmental trait. The heritability estimate of number of services per conception was low in the present study (0.042±0.003). The low estimate implied that genetics have some scope for improvement in the trait, but the management practices followed in the herd control the trait. Therefore, care should be taken in estrus detection and timing of insemination, semen quality assessment, and better nutritional status of the animal to improve the trait. As seen in the present study, lower estimates of heritability for reproduction traits were obtained in other breeds of cattle [17,18].

#### Repeatability

There are only a few studies on repeatability in Jersey crossbred cattle. Repeatability is the fraction of the variance that is attributed to permanent differences between individuals. Higher the repeatability, more accurate will be the culling of cows on the basis of the first lactation. In the present study, repeatability values of the service period, dry period, and calving interval were lower than those of the earlier report of Khan *et al*. [19] in Jersey × Red Sindhi. However, the estimated values are higher than those reports in Jersey × Sahiwal crossbreds [16]. The estimates were low to moderate in magnitude. The low repeatability estimates of reproduction traits ranged from 0.001 (number of services per conception) to 0.42 (service period) indicate that they were influenced more by temporary environmental effects.

## Conclusions

The reproduction performances of Jersey × Red Sindhi crossbreds were low when compared to the earlier reports on other Jersey crossbreds. From this study, we concluded that lifetime profitability could be increased by an earlier age at first calving and shorter calving interval under uniform feeding and management conditions. Low to moderate values of heritability for reproductive traits indicated limited scope for improvement through selection.

## Authors’ Contributions

SV executed the work (collection of data, analysis, and writing of manuscript); AS, CJ, and SNS planned the study and formulated the experimental design; RV helped in the analysis of data and writing of the manuscript; All authors read and approved the final manuscript.
